# Supplementary material for: In-degree centrality in a social network is linked to coordinated neural activity
Source: Nat Commun. 2022 Mar 2;13:1118. doi: 10.1038/s41467-022-28432-3 (PMC8891270; doi:10.1038/s41467-022-28432-3)
Supplement: Supplementary file 1 — Supplementary Information [file 41467_2022_28432_MOESM1_ESM.docx]

Supplementary Information for **“In-degree centrality in a social network is linked to coordinated neural activity”**

Elisa C. Baek^1^*, Ryan Hyon^1^, Karina López^1^, Emily S. Finn^2^, Mason A. Porter^3,4^, and Carolyn Parkinson^1,5^*

^1^Department of Psychology, University of California, Los Angeles, Los Angeles, CA, United States of America, ^2^Department of Psychological and Brain Sciences, Dartmouth College, Hanover, NH, United States of America, ^3^Department of Mathematics, University of California, Los Angeles, Los Angeles, CA, United States of America, ^4^Sante Fe Institute, Santa Fe, NM, United States of America, ^5^Brain Research Institute, University of California, Los Angeles, Los Angeles, CA, United States of America

* Corresponding authors

**Supplementary Fig. 1: Distributions of in-degree centrality**

**
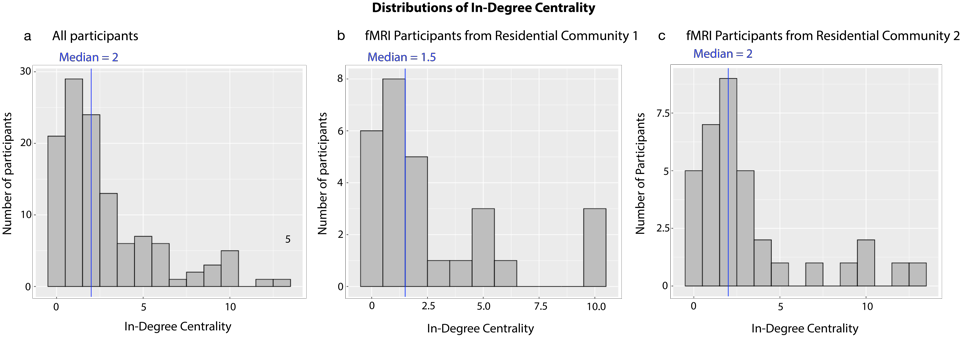
**

**Supplementary Fig. 1. Distributions of in-degree centrality.** The distributions of in-degree centrality are relatively similar across the two residential communities. (a) The distribution of in-degree centrality for the set of all participants who participated in our social-network survey. (b) The distribution of in-degree centrality in the subset of the fMRI participants who were living in residential community 1. (c) The distribution of in-degree centrality in the subset of the fMRI participants who were living in residential community 2.

**Supplementary Table 1: Stimuli descriptions**

Supplementary Table 1. Descriptions of stimuli

| Video # | Run # | Video | Content |
| --- | --- | --- | --- |
| 1 | 1 | An Astronaut’s View of Earth | An astronaut discusses viewing Earth from space and, in particular, witnessing the effects of climate change from space. He then urges viewers to mobilize to address this issue. |
| 2 | 1 | All I Want | A sentimental music video depicting a social outcast with a facial deformity who is seeking companionship. |
| 3 | 1 | Scientific demonstration | An astronaut at the International Space Station demonstrates and explains what happens when one wrings out a waterlogged washcloth in space. |
| 4 | 1 | Food Inc. | An excerpt from a documentary discussing how the fast-food industry influences food production and farming practices in the United States. |
| 5 | 2 | We Can Be Heroes | An excerpt from a mockumentary-style series in which a man discusses why he nominated himself for the title of Australian of the Year. |
| 6 | 2 | Ban College Football | Journalists and athletes debate whether football should be banned as a college sport. |
| 7 | 2 | Soccer match | Highlights from a soccer match. |
| 8 | 2 | Ew! | A comedy skit in which grown men play teenage girls disgusted by the things around them. |
| 9 | 2 | Life’s Too Short | An example of a ‘cringe comedy’ in which a dramatic actor is depicted unsuccessfully trying his hand at improvisational comedy. |
| 10 | 2 | America’s Funniest Home Videos | A series of homemade video clips that depict examples of unintentional physical comedy arising from accidents. |
| 11 | 3 | Zima Blue | A philosophical, animated short set in a futuristic world. |
| 12 | 3 | Nathan For You | An episode from a ‘docu-reality’ comedy in which the host convinces people, who are not always in on the joke, to engage in a variety of strange behaviors. |
| 13 | 4 | College Party | An excerpt from a film that depicts a party scene in which a bashful college student is pressured to drink alcohol. |
| 14 | 4 | Eighth Grade | Two excerpts from a film. They depict (1) a young teenager who video blogs about her mental-health issues and (2) an awkward scene between two teenagers on a dinner date. |

Note: Videos 1–10 are a subset of the videos that were used in a prior study^1^. The descriptions of them in the present paper are the same as those in the prior study.

**Supplementary Table 2 and Supplementary Table 3 for participant-level ISC analysis: Participant-level subcortical results**

Supplementary Table 2. Participant-level results that relate ISCs with the binarized in-degree centrality

(high versus low): Subcortical results

| Subcortical region | β | SE | | *p*  (corrected) | *p*  (uncorrected) |
| --- | --- | --- | --- | --- | --- |
| Nucleus accumbens (L) | 0.363 | | 0.260 | 0.325 | 0.167 |
| Amygdala (L) | 0.578 | | 0.253 | 0.164 | 0.026 |
| Caudate nucleus (L) | 0.269 | | 0.262 | 0.466 | 0.307 |
| Hippocampus (L) | 0.550 | | 0.254 | 0.180 | 0.031 |
| Pallidum (L) | 0.611 | | 0.252 | 0.144 | 0.018 |
| Putamen (L) | 0.300 | | 0.261 | 0.415 | 0.256 |
| Thalamus (L) | 0.432 | | 0.258 | 0.247 | 0.099 |
| Nucleus accumbens (R) | 0.080 | | 0.264 | 0.834 | 0.764 |
| Amygdala (R) | 0.493 | | 0.256 | 0.210 | 0.059 |
| Caudate nucleus (R) | 0.171 | | 0.263 | 0.640 | 0.519 |
| Hippocampus (R) | 0.587 | | 0.253 | 0.158 | 0.024 |
| Pallidum (R) | 0.068 | | 0.264 | 0.845 | 0.798 |
| Putamen (R) | 0.302 | | 0.261 | 0.414 | 0.252 |
| Thalamus (R) | 0.482 | | 0.256 | 0.220 | 0.065 |

We have FDR-corrected all *p*-values because of multiple comparisons; we also report the corresponding uncorrected *p-*values. All of the reported *p*-values are two-tailed. The quantity β is the standardized regression coefficient, and the quantity SE is the standard error.

Supplementary Table 3. Participant-level results that relate ISCs with the original (i.e., non-binarized) in-degree centrality: Subcortical results

| Subcortical region | *ρ* | *p*  (corrected) | | *p*  (uncorrected) |
| --- | --- | --- | --- | --- |
| Nucleus accumbens (L) | 0.248 | | 0.217 | 0.050 |
| Amygdala (L) | 0.350 | | 0.104 | 0.005 |
| Caudate nucleus (L) | 0.167 | | 0.355 | 0.191 |
| Hippocampus (L) | 0.302 | | 0.175 | 0.016 |
| Pallidum (L) | 0.256 | | 0.217 | 0.043 |
| Putamen (L) | 0.123 | | 0.491 | 0.337 |
| Thalamus (L) | 0.212 | | 0.262 | 0.096 |
| Nucleus accumbens (R) | 0.054 | | 0.787 | 0.673 |
| Amygdala (R) | 0.217 | | 0.262 | 0.088 |
| Caudate nucleus (R) | 0.063 | | 0.742 | 0.624 |
| Hippocampus (R) | 0.279 | | 0.200 | 0.027 |
| Pallidum (R) | –0.003 | | 0.989 | 0.981 |
| Putamen (R) | 0.154 | | 0.392 | 0.227 |
| Thalamus (R) | 0.251 | | 0.217 | 0.047 |

We have FDR-corrected all *p*-values because of multiple comparisons; we also report the

corresponding uncorrected *p*-values. All of the reported *p*-values are two-tailed.

**Supplementary Fig. 2 for participant-level ISC analysis: Scatterplots of the relationships between in-degree centrality and mean neural similarity in key brain regions**

**
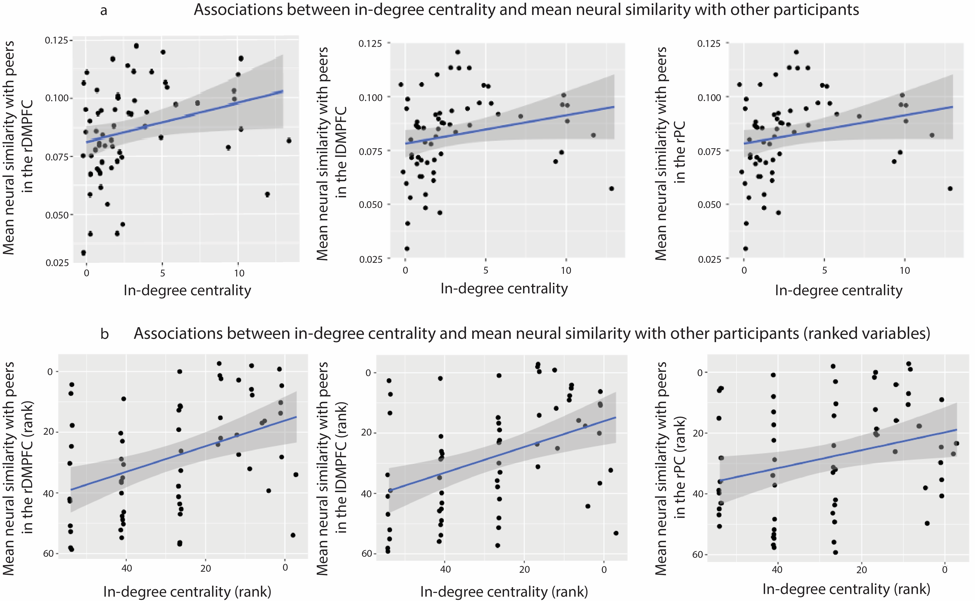
**

**Supplementary Fig. 2. Scatter plots of the relationships between in-degree centrality and mean neural similarity of participants in key brain regions.** (a) The relationships between in-degree centrality and mean neural similarity in the rDMPFC, lDMPFC, and rPC. (b) The relationships between ranked in-degree centrality and ranked mean neural similarity in the rDMPFC, lDMPFC, and rPC.

**Supplementary Methods 1 for participant-level ISC analysis**

**Binarized participant-level results that control for similarities in self-reported demographic traits.** We fit analogous models to those that we described in the participant-level ISC analysis in the Results section of the main manuscript to test the relationship between in-degree centrality and ISC while controlling for all available self-reported demographic variables: similarities in age, gender, ethnicity, and home country (which we define as the country where an individual was living prior to enrolling at the university). To control for similarities in demographic variables, for each unique dyad (i.e., for each pair of individuals) who participated in the fMRI session, we computed an absolute difference of the age between each individual in the dyad (i.e., age_difference = |age_1_ – age_2_|). We then transformed this difference score into a similarity score such that larger numbers indicated greater similarity (specifically, age_similarity = 1 – (age_difference/max(age_difference)). To control for similarities in gender, we created an indicator variable in which 0 signifies different genders and 1 signifies the same gender. To control for similarities in ethnicity and race, we created an indicator variable for each ethnicity category (Asian, Black/African, Hispanic/Latinx, Native American, Pacific Islander, and Caucasian/White) in which 0 signifies a different self-reported ethnicity and 1 signifies the same self-reported ethnicity. Participants were able to self-report as many ethnicities as they desired. For each unique dyad, we used an overall indicator variable for ethnicity in which 0 signifies no shared ethnicity and 1 signifies a shared ethnicity. If two members of a dyad self-reported even one same ethnicity, we coded them as having a shared ethnicity. To control for similarities in home country, we used an additional indicator variable in which 0 signifies different home countries and 1 signifies the same home country. For each participant, we then calculated the mean similarity between them and each other participant for each of the demographic variables and used these variables as covariates in our mixed-effects models. These models gave a similar pattern of results as those that we reported in the main manuscript (see Supplementary Fig. 3).

**Supplementary Figure 3 for participant-level ISC analysis**

**
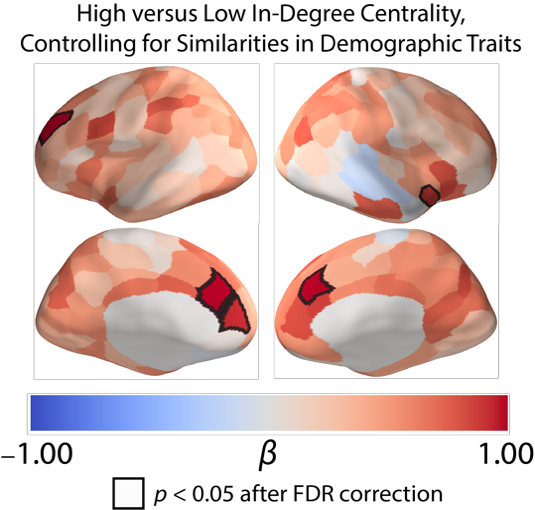
**

**Supplementary Fig. 3. Participant-level results that control for similarities in demographic traits (specifically, for mean similarities with other participants in age, gender, ethnicity and race, and home country).** The association between in-degree centrality and ISCs in the bilateral DMPFC that we reported in the main manuscript remains significant after controlling for similarities in demographic traits. The quantity β is the standardized regression coefficient. Regions where we observed significant associations between in-degree centrality and ISC are outlined in black. We used an FDR-corrected significance threshold of *p* < 0.05, which corresponds to an uncorrected *p*-value threshold of *p* < 0.001. All of the reported *p*-values are two-tailed.

**Supplementary Methods 2 for participant-level ISC analysis**

**Binarized participant-level results: Intra-community ISC only.** Because our participants came from two different residential communities, we also conducted analyses to test if we could observe similar patterns in associations between ISCs and in-degree centrality while only using intra-community ISCs. To do this, we fit analogous modaels as to those that we described in the participant-level ISC analysis in the Results section of the main manuscript, except that we calculated each participant’s mean neural similarity in each brain region using only other members of their own residential community. That is, we removed dyads in which both members did not belong to the same community. The results of these calculations (see Supplementary Fig. 4) are similar to those that we reported in the main manuscript.

**Supplementary Fig. 4 for participant-level ISC analysis**


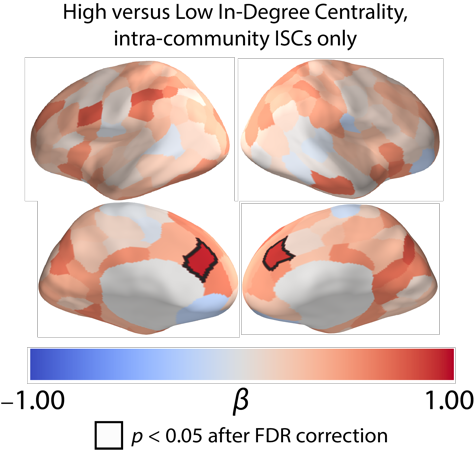


**Supplementary Fig. 4. Participant-level results for intra-community ISCs only.** The results of our calculations using only intra-community ISCs identified a similar pattern of results (specifically, large ISCs in the bilateral DMPFC) as in our main analysis (see Fig. 2) as significantly associated with in-degree centrality. The quantity β is the standardized regression coefficient. Regions where we observed significant associations between in-degree centrality and ISC are outlined in black. We used an FDR-corrected significance threshold of *p* < 0.05, which corresponds to an uncorrected *p*-value threshold of *p* < 0.001. All of the reported *p*-values are two-tailed.

**Supplementary Methods 3 for participant-level ISC analysis**

**Binarized participant-level results: Intra-community ISCs only that control for self-reported demographic traits and social distances.** We also conducted analyses to test if the associations between ISCs and in-degree centrality remain significant after we control both for demographic variables (specifically, for mean similarities with community members in age, gender, ethnicity and race, and home country) and for the social distances between individuals, given that social distance has been associated previously with neural similarities^1,2^. (See Supplementary Methods 1 above for details of how we calculated similarities in age, gender, ethnicity and race, and home country.) To do this, we fit GLMs to infer ISCs in each brain region from in-degree centrality while including all of the control variables as covariates in the model. For these computations, we used ISCs in intra-community dyads only, so we calculated each individual’s mean ISC based on their ISCs only with other members of their own residential community. For each individual, we took the following steps to calculate their mean social distance from each other participant from their community. For each intra-community dyad, we defined “social distance” to be the smallest number of intermediate social ties (i.e., the geodesic distance) that is necessary to connect the two individuals in the network via a path. We calculated social distances using an unweighted and undirected network that includes a tie between two individuals even if only one of them nominated the other as a friend. We set the social distance between any pair of individuals who were on different components of the network as equal to 1 more than the maximum measured social distance in the network. In both of the residential communities, the maximum measured social distance was 6, so we coded such individuals who were on different components of the network to have a social distance of 7 between them. In our participant-level analysis, for each individual, we took the mean social distance between them and each other member of their residential community and used this variable as a covariate in our GLM for inferring ISCs. The results of these calculations (see Supplementary Fig. 5) are similar to those that we reported in the main manuscript.

**Supplementary Fig. 5 for participant-level ISC analysis**

**
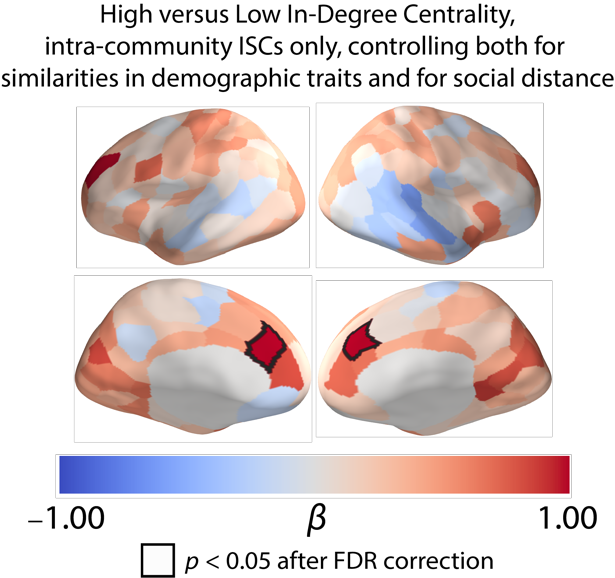
**

**Supplementary Fig. 5. Participant-level results for intra-community ISCs only when controlling both for similarities in demographic traits and for social distances.** We again identified a similar pattern of results (specifically, large ISCs in the bilateral DMPFC) as in our main analysis (see Fig. 2) as significantly associated with in-degree centrality. The quantity β is the standardized regression coefficient. Regions where we observed significant associations between in-degree centrality and ISC are outlined in black. We used an FDR-corrected significance threshold of *p* < 0.05. which corresponds to an uncorrected *p*-value threshold of *p* < 0.001. All of the reported *p*-values are two-tailed.

**Supplementary Methods 4 for participant-level ISC analysis**

**Binarized participant-level results with approximately equal-sized groups.** Because our median-split approach yields unequally sized in-degree centrality groups (*n*_high_ = 23; *n*_low_ = 40), we also tested associations between ISCs and the binarized in-degree centrality variable with approximately equal-sized groups. To do so, we took a subset of the data by removing all participants with the median in-degree centrality value (specifically, with an in-degree centrality equal to 2). We then classified participants as part of the high-centrality group if they had an in-degree that was larger than the median (specifically, if it was more than 2; there were *n*_high_ = 23 such people) and into the low-centrality group if they had an in-degree that was less than the median (specifically, if it was less than 2; there were *n*_low_ = 26 such people). Similar to our main participant-level results, relationships between ISCs in the right and left DMPFC and in-degree centrality were significant even when using this subset of the data. We also found that the ISCs in other regions (including the precuneus and the inferior parietal lobule) of the default mode network were also associated with in-degree centrality (see Supplementary Fig. 6).

**Supplementary Fig. 6 for participant-level ISC analysis**


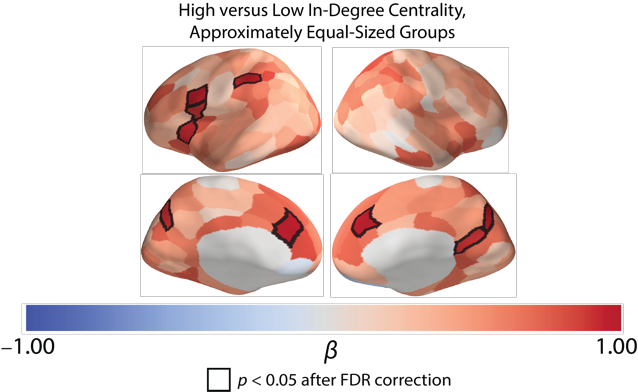


**Supplementary Fig. 6. Participant-level results with approximately equal-sized in-degree centrality groups.** The associations between ISCs in the right and left DMPFC and in-degree centrality remain significant when we used a subset of the data with approximately equal-sized in-degree centrality groups. Additionally, we found that the ISCs in other regions (including the precuneus and the inferior parietal lobule) of the default mode network were also associated with in-degree centrality. The quantity β is the standardized regression coefficient. Regions where we observed significant associations between in-degree centrality and ISC are outlined in black. We used an FDR-corrected significance threshold of *p* < 0.05, which corresponds to an uncorrected *p*-value threshold of *p* < 0.002. All of the reported *p*-values are two-tailed.

**Supplementary Methods 5 for participant-level ISC analysis**

**Binarized participant-level results that control for preference similarities.** The relationships between in-degree centrality and the ISCs in the right and left DMPFC remain significant after controlling for similarities in enjoyment and interest ratings. This suggests that neural similarities in these regions capture similarities beyond those that we observed from self-reported preference ratings (see Supplementary Fig. 7).

**Supplementary Fig. 7 for participant-level ISC analysis**


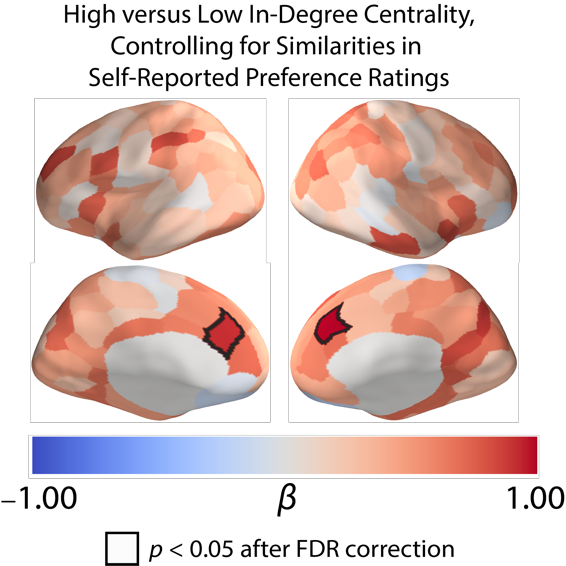


**Supplementary Fig. 7. Participant-level results that control for similarities in self-reported preference ratings.** The associations between in-degree centrality and ISCs in the right and left DMPFC and remain significant after controlling for similarities in self-reported preference ratings (i.e., ratings of enjoyment of and interest in stimuli). The quantity β is the standardized regression coefficient. Regions where we observed significant associations between in-degree centrality and ISC are outlined in black. We used an FDR-corrected significance threshold of *p* < 0.05, which corresponds to an uncorrected *p*-value threshold of *p* < 0.001. All of the reported *p*-values are two-tailed.

**Supplementary Tables 4–6 for dyad-level ISC analysis: Subcortical results**

Supplementary Table 4. Dyad-level results that relate ISCs with binarized in-degree centrality

Contrast: ISC_{high, high}_ > ISC_{low, low}_

| Subcortical region | β | SE | | *p*  (corrected) | *p*  (uncorrected) |
| --- | --- | --- | --- | --- | --- |
| Nucleus accumbens (L) | 0.194 | | 0.120 | 0.054 | 0.022 |
| Amygdala (L) | 0.505 | | 0.218 | 0.007* | 0.001 |
| Caudate nucleus (L) | 0.193 | | 0.174 | 0.190 | 0.117 |
| Hippocampus (L) | 0.448 | | 0.208 | 0.012* | 0.002 |
| Pallidum (L) | 0.263 | | 0.109 | 0.005** | < 0.001 |
| Putamen (L) | 0.185 | | 0.160 | 0.168 | 0.107 |
| Thalamus (L) | 0.218 | | 0.146 | 0.078 | 0.035 |
| Nucleus accumbens (R) | –0.026 | | 0.114 | 0.795 | 0.749 |
| Amygdala (R) | 0.402 | | 0.210 | 0.024* | 0.007 |
| Caudate nucleus (R) | 0.105 | | 0.172 | 0.466 | 0.385 |
| Hippocampus (R) | 0.525 | | 0.213 | 0.004** | < 0.001 |
| Pallidum (R) | 0.020 | | 0.094 | 0.807 | 0.763 |
| Putamen (R) | 0.242 | | 0.175 | 0.103 | 0.052 |
| Thalamus (R) | 0.294 | | 0.171 | 0.041* | 0.015 |

* *p* < 0.05, ** *p* < 0.01; we have FDR-corrected all *p*-values because of multiple comparisons.

We also report the corresponding uncorrected *p*-values. All of the reported *p*-values are two-tailed.

Supplementary Table 5. Dyad-level results that relate ISCs with binarized in-degree centrality

Contrast: ISC_{high, high}_ > ISC_{low, high}_

| Subcortical region | β | SE | | *p*  (corrected) | *p*  (uncorrected) |
| --- | --- | --- | --- | --- | --- |
| Nucleus accumbens (L) | 0.151 | | 0.011 | 0.034* | 0.011 |
| Amygdala (L) | 0.268 | | 0.002 | 0.009** | 0.002 |
| Caudate nucleus (L) | 0.123 | | 0.092 | 0.156 | 0.092 |
| Hippocampus (L) | 0.221 | | 0.007 | 0.024* | 0.007 |
| Pallidum (L) | 0.139 | | 0.015 | 0.041* | 0.015 |
| Putamen (L) | 0.099 | | 0.151 | 0.232 | 0.151 |
| Thalamus (L) | 0.064 | | 0.331 | 0.413 | 0.331 |
| Nucleus accumbens (R) | –0.120 | | 0.039 | 0.085 | 0.039 |
| Amygdala (R) | 0.197 | | 0.017 | 0.046* | 0.017 |
| Caudate nucleus (R) | 0.043 | | 0.551 | 0.625 | 0.551 |
| Hippocampus (R) | 0.319 | | 0.000 | 0.002** | < 0.001 |
| Pallidum (R) | 0.004 | | 0.941 | 0.951 | 0.941 |
| Putamen (R) | 0.192 | | 0.009 | 0.029* | 0.009 |
| Thalamus (R) | 0.101 | | 0.162 | 0.241 | 0.162 |

* *p* < 0.05, ** *p* < 0.01; we have FDR-corrected all *p*-values because of multiple comparisons.

We also report the corresponding uncorrected *p*-values. All of the reported *p*-values are two-tailed.

Supplementary Table 6. Dyad-level results that relate ISCs with binarized in-degree centrality

Contrast: ISC_{low, high}_ > ISC_{low, low}_

| Subcortical region | β | SE | | *p*  (corrected) | *p*  (uncorrected) |
| --- | --- | --- | --- | --- | --- |
| Nucleus accumbens (L) | 0.043 | | 0.365 | 0.448 | 0.364 |
| Amygdala (L) | 0.237 | | 0.003 | 0.013* | 0.003 |
| Caudate nucleus (L) | 0.070 | | 0.282 | 0.367 | 0.282 |
| Hippocampus (L) | 0.226 | | 0.003 | 0.014* | 0.003 |
| Pallidum (L) | 0.124 | | 0.006 | 0.022* | 0.006 |
| Putamen (L) | 0.086 | | 0.153 | 0.233 | 0.153 |
| Thalamus (L) | 0.154 | | 0.006 | 0.022* | 0.006 |
| Nucleus accumbens (R) | 0.094 | | 0.041 | 0.088 | 0.041 |
| Amygdala (R) | 0.205 | | 0.008 | 0.026* | 0.008 |
| Caudate nucleus (R) | 0.062 | | 0.332 | 0.413 | 0.332 |
| Hippocampus (R) | 0.206 | | 0.008 | 0.027* | 0.008 |
| Pallidum (R) | 0.016 | | 0.692 | 0.746 | 0.692 |
| Putamen (R) | 0.050 | | 0.445 | 0.518 | 0.445 |
| Thalamus (R) | 0.193 | | 0.003 | 0.012* | 0.003 |

* *p* < 0.05, ** *p* < 0.01; we have FDR-corrected all *p*-values because of multiple comparisons. We also report the corresponding uncorrected *p*-values. All of the reported *p*-values are two-tailed.

**Supplementary Methods 6 for Dyad-level ISC analysis**

**Binarized dyad-level ISC results that control for similarities in demographic traits and friendships.** We also tested whether our findings remain significant after controlling for dyadic similarities in demographic traits (specifically, similarities in age, gender, ethnicity and race, and home country) and whether or not a given pair of participants were friends with each other. (See Supplementary Methods 1 above for descriptions of how we computed similarities in age, gender, ethnicity and race, and home country.) If either individual in a dyad nominated the other as a friend, we coded the dyad as signifying an undirected friendship. We controlled for friendship, rather than social distance, because our primary analyses included ISCs between individuals from different residential communities. In Supplementary Methods 7 below, we discuss analyses that are based only on intra-community dyads that also control for social distances between individuals in a dyad. Our results indicate that our pattern of results that links ISC and dyad-level in-degree centrality remains significant after we control for similarities in demographic variables and friendship (see Supplementary Fig. 8). This suggests that our findings that the greatest neural similarity occurs between highly-central individuals and that lower neural similarity occurs between less-central individuals arose from differences beyond what we captured using similarities in demographic traits and friendships between individuals in the same dyad.

**Supplementary Fig. 8 for dyad-level ISC analysis**


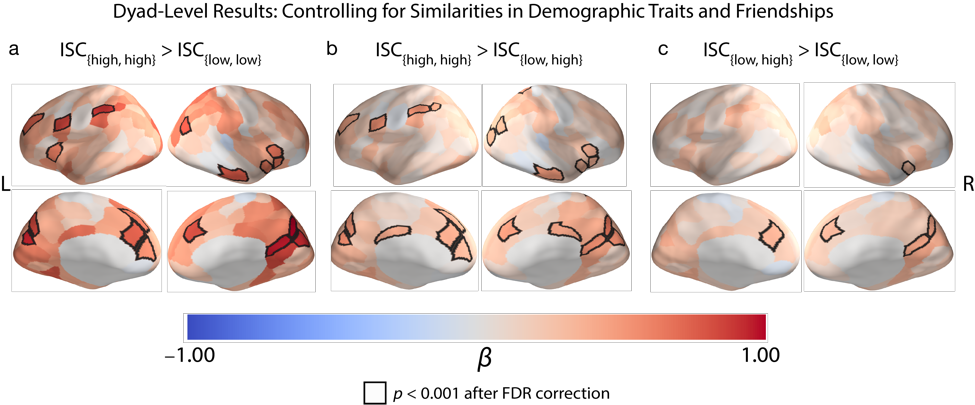


**Supplementary Fig. 8. Dyad-level associations of neural similarity with in-degree centrality when controlling for similarities in demographic traits and friendships.** We identified similar brain regions as significantly associated with in-degree centrality as in our results in the main manuscript (see Fig. 4). The quantity β is the standardized regression coefficient. Regions where we observed significant associations between in-degree centrality and ISC are outlined in black. We used an FDR-corrected significance threshold of *p* < 0.001, which corresponds to an uncorrected *p*-value threshold of *p* < 1.947 × 10^-6^. All of the reported *p*-values are two-tailed.

**Supplementary Methods 7 for dyad-level ISC analysis**

**Binarized dyad-level results: Intra-community ISCs only.** Because our participants came from two different residential communities, we also conducted analyses to test if our findings remain significant when we used only intra-community ISCs. To do this, we fit analogous models to those that we described in dyad-level ISC analysis in the Results section of the main manuscript to test for associations between ISCs and dyad-level in-degree centrality, except that we calculated each subject’s mean neural similarity in each brain region based only on other members of their own residential community. (That is, we removed dyads with individuals that did not belong to the same community.) We obtained similar results (see Supplementary Fig. 9) as the ones that we reported in the main manuscript.

**Supplementary Fig. 9 for dyad-level ISC analysis**


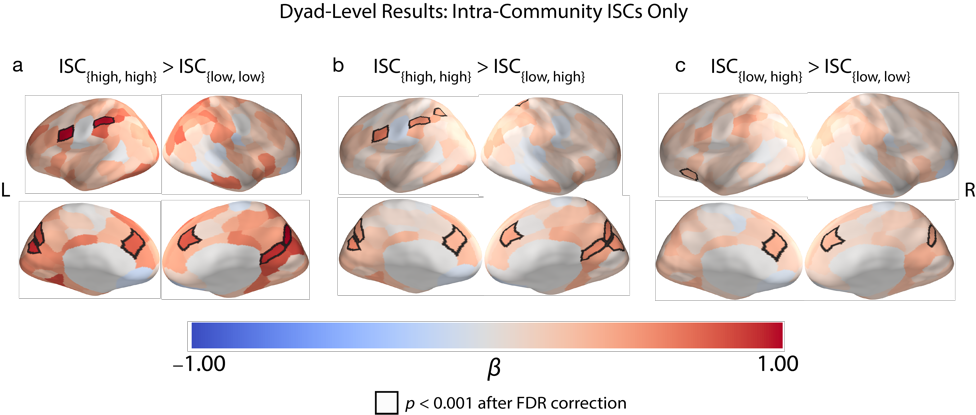


**Supplementary Fig. 9. Dyad-level associations of neural similarity with in-degree centrality when we used intra-community ISCs only.** We identified similar brain regions as significantly associated with in-degree centrality as in our results in the main manuscript (see Fig. 4). The quantity β is the standardized regression coefficient. Regions where we observed significant associations between in-degree centrality and ISC are outlined in black. We used an FDR-corrected significance threshold of *p* < 0.001, which corresponds to an uncorrected *p*-value threshold of *p* < 3.738 × 10^-5^. All of the reported *p*-values are two-tailed.

**Supplementary Methods 8 for dyad-level ISC analysis**

**Binarized dyad-level results: Intra-community ISCs only when controlling both for demographic variables and for social distances.** We also tested if our results remain significant after controlling both for demographic variables (specifically, for mean similarities with community members in age, gender, ethnicity and race, and home country) and for social distances between individuals. (See Supplementary Methods 1 above for descriptions of how we computed similarities in age, gender, ethnicity and race, and home country.) To do this, we fit mixed-effects models that infer ISCs in each brain region from the dyad-level in-degree centrality variable while including all of the control variables as covariates. For these analyses, we examined ISCs in intra-community dyads only, so we included only data from individuals from the same residential community. We defined social distance as we described in Supplementary Methods 6, and we included the social distance between the individuals of each dyad as a covariate in our mixed-effects models for inferring ISC in each brain region from the dyad-level in-degree centrality variable. We obtained results (see Supplementary Fig. 10) that were similar to those that we reported in the main manuscript. This suggests that our finding that neural similarity is linked with in-degree centrality is not merely a confound of the most-popular individuals being more likely to be friends with one another.

**Supplementary Fig. 10 for dyad-level ISC analysis**


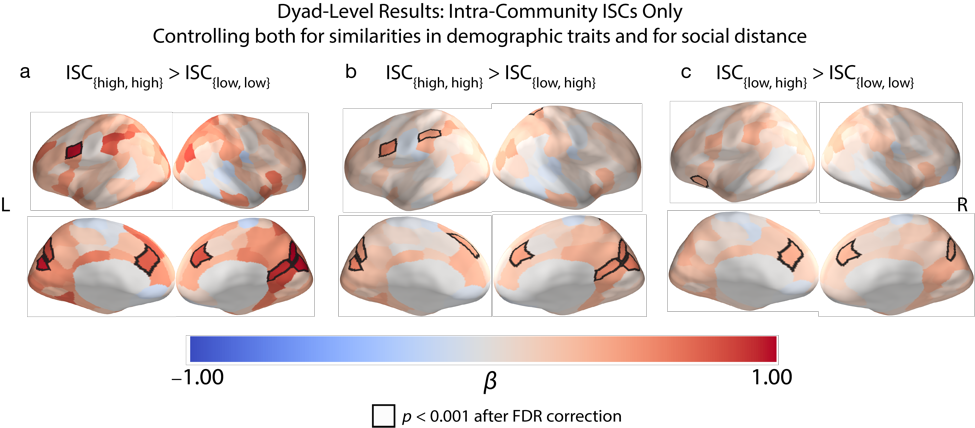


**Supplementary Fig. 10. Dyad-level associations of neural similarity with in-degree centrality for intra-community ISCs only when controlling both for similarities in demographic traits and for social distances between individuals in a dyad.** We identified similar brain regions as significantly associated with in-degree centrality as in our results in the main manuscript (see Fig. 4). The quantity β is the standardized regression coefficient. Regions where we observed significant associations between in-degree centrality and ISC are outlined in black. We used an FDR-corrected significance threshold of *p* < 0.001, which corresponds to an uncorrected *p*-value threshold of *p* < 3.583 × 10^-5^. All of the reported *p*-values are two-tailed.

**Supplementary Methods 9 for dyad-level ISC analysis**

**Binarized dyad-level results with approximately equal-sized groups.** Because our median-split approach yields unequally sized in-degree centrality groups, we also tested the associations between ISCs and the binarized in-degree centrality variable with approximately equal-sized groups of individuals (see Supplementary Methods 4). We took an analogous approach to one that we discussed in the main manuscript (see the Results and Methods sections for more details) to transform the participant-level in-degree centrality measure into a dyad-level measure. Of the 1175 unique dyads, 324 of them were {high, high}, 253 of them were {low, low}, and 598 of them were {low, high}. We obtained results (see Supplementary Fig. 11) that were similar to those that we reported in the main manuscript.

**Supplementary Fig. 11 for dyad-level ISC analysis**

**
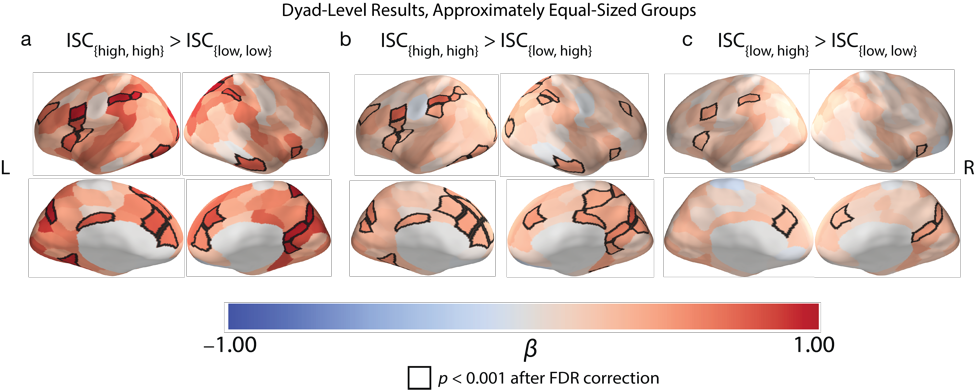
**

**Supplementary Fig. 11. Dyad-level associations of neural similarity with in-degree centrality with approximately equal-sized in-degree centrality groups.** We identified similar brain regions as significantly associated with in-degree centrality as in our results in the main manuscript (see Fig. 4) when we used a subset of the data with approximately equal-sized centrality groups. The quantity β is the standardized regression coefficient. Regions where we observed significant associations between in-degree centrality and ISC are outlined in black. We used an FDR-corrected significance threshold of *p* < 0.001, which corresponds to an uncorrected *p*-value threshold of *p* < 0.0001. All of the reported *p*-values are two-tailed.

**Supplementary Methods 9 for dyad-level preference analysis**

Our findings in the main text suggest that self-reported preference data are consistent with our hypothesis that highly-central individuals have preferences that are exceptionally similar to each other and less-central individuals have preferences that are idiosyncratic, with each low-centrality individual’s preferences differing from those of other individuals in their own way. See Supplementary Tables 7 and 8.

**Supplementary Table 7 and Supplementary Table 8 for dyad-level preference analysis**

Table S7. Inferring similarities in enjoyment ratings from dyad-level in-degree centralities

| Contrast | β | | SE | *p*  (corrected) | *p*  (uncorrected) |
| --- | --- | --- | --- | --- | --- |
| *s*_{high,high}_ > *s*_{low,low}_ | 0.589 | 0.251 | | 0.001** | 0.0009 |
| *s*_{high,high}_ > *s*_{low,high}_ | 0.322 | 0.133 | | 0.001** | 0.0007 |
| *s*_{low,high}_ > *s*_{low,low}_ | 0.267 | 0.133 | | 0.003** | 0.003 |

* *p* < 0.05, ** *p* < 0.01; we have FDR-corrected all *p*-values because of multiple comparisons;

we also report the corresponding uncorrected *p*-values. All of the reported *p*-values are two-tailed.

Table S8. Inferring similarities in interest ratings from dyad-level in-degree centralities

| Contrast | β | | SE | *p*  (corrected) | *p*  (uncorrected) |
| --- | --- | --- | --- | --- | --- |
| *s*_{high,high}_ > *s*_{low,low}_ | 0.509 | 0.259 | | 0.008** | 0.005 |
| *s*_{high,high}_ > *s*_{low,high}_ | 0.277 | 0.137 | | 0.008** | 0.004 |
| *s*_{low,high}_ > *s*_{low,low}_ | 0.232 | 0.137 | | 0.013* | 0.013 |

* *p* < 0.05, ** *p* < 0.01; we have FDR-corrected all *p*-values because of multiple comparisons;

we also report the corresponding uncorrected *p*-values. All of the reported *p*-values are two-tailed.

**Supplementary Methods 10 for dyad-level preference analysis**

**Binarized dyad-level ISC results: Controlling for similarities in preference.** We tested whether our findings that individuals who were highly central in their social network tended to have exceptionally similar neural responses to other highly-central individuals and that less-central individuals had idiosyncratic responses could be attributable to inter-subject similarities in self-reported preferences. Although similarities in interest and enjoyment ratings were associated with neural similarity in subregions of the default mode network (see Supplementary Fig. 12), when we controlled for dyad-level binarized in-degree centrality by including it as a covariate, we found that the associations between similarities in preference ratings and ISCs in many of the brain regions were no longer significant (see Supplementary Fig 13). Notably, we also found that the pattern of results that link ISCs and dyad-level binarized in-degree centrality remain significant after controlling for similarity in enjoyment and interest ratings (see Supplementary Fig. 14). This suggests that our findings that the greater neural similarity occurs between highly-central individuals and that less neural similarity occurs between less-central individuals resulted from differences beyond what we captured using self-reported preference ratings.

**Supplementary Fig. 12 for dyad-level preference analysis**


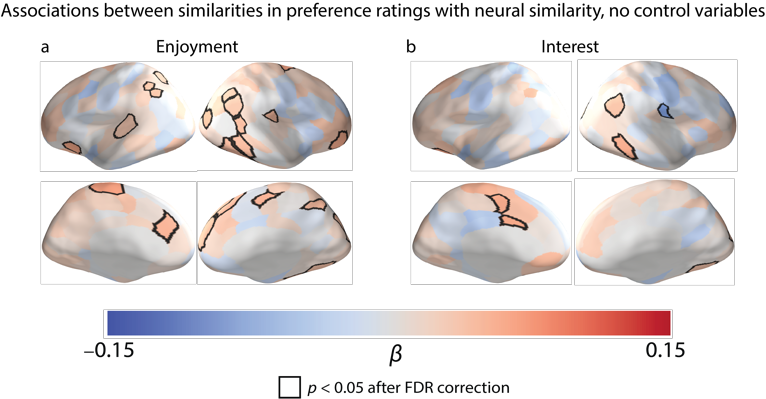


**Supplementary Fig. 12. Dyad-level associations of neural similarity with similarities in preference ratings.** We found that similarities in (a) interest and (b) enjoyment ratings were associated with neural similarity in regions of the default mode network when not controlling for in-degree centrality. The quantity β is the standardized regression coefficient. Regions where we observed significant associations between similarities in enjoyment (a) and interest (b) ratings and ISC are outlined in black. We used an FDR-corrected significance threshold of *p* < 0.05, which corresponds to an uncorrected *p*-value threshold of *p* < 0.006 for (a) and *p* < 0.002 for (b). All of the reported *p*-values are two-tailed.

**Supplementary Fig. 13 for dyad-level preference analysis**


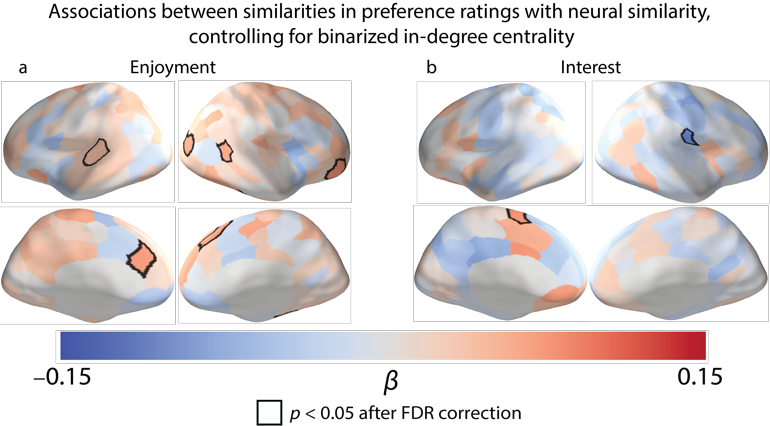


**Supplementary Fig. 13. Dyad-level associations of neural similarity with similarities in preference ratings when controlling for in-degree centrality.** We found that the associations between similarities in preference ratings and ISCs in many of the regions were no longer significant when we controlled for dyad-level binarized in-degree centrality. The quantity β is the standardized regression coefficient. Regions where we observed significant associations between similarities in enjoyment (a) and interest (b) ratings and ISC are outlined in black. We used an FDR-corrected significance threshold of *p* < 0.05, which corresponds to an uncorrected *p*-value threshold of *p* < 0.002 for (a) and *p* < 0.001 for (b). All of the reported *p*-values are two-tailed.

**Supplementary Fig. 14 for dyad-level preference analysis**


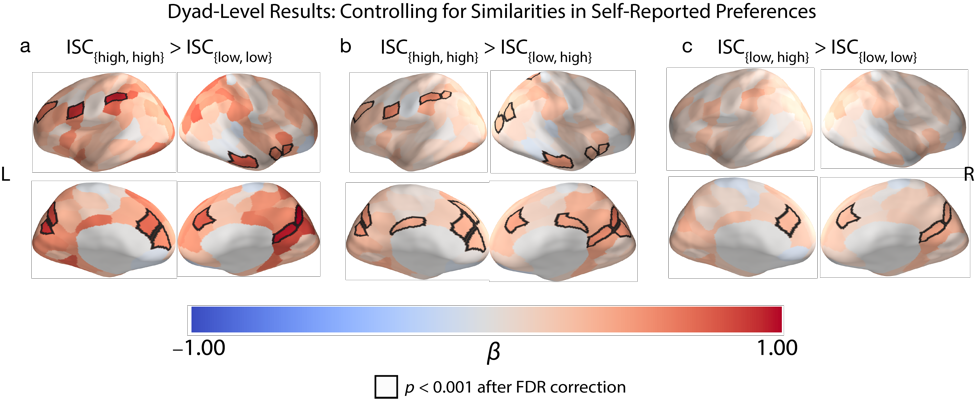


**Supplementary Fig. 14. Dyad-level associations of neural similarity with in-degree centrality when controlling for similarities in self-reported preferences (specifically, interest and enjoyment ratings) about the videos.** We identified similar brain regions as significantly associated with in-degree centrality as in our main manuscript (see Fig. 4). The quantity β is the standardized regression coefficient. Regions where we observed significant associations between in-degree centrality and ISC are outlined in black. We used an FDR-corrected significance threshold of *p* < 0.001, which corresponds to an uncorrected *p*-value threshold of *p* < 6.075 × 10^-5^. All of the reported *p*-values are two-tailed.

**Supplementary Table 9: Table of excluded participants**

| Participant | Reason(s) |
| --- | --- |
| Excluded participant 1 | Excessive movement during the fMRI scan |
| Excluded participant 2 | Excessive movement during the fMRI scan |
| Excluded participant 3 | Fell asleep during half of the fMRI scan |
| Excluded participant 4 | Did not complete the fMRI scan and did not complete the social-network survey |
| Excluded participant 5 | Did not complete the social-network survey |
| Excluded participant 6 | Did not complete the social-network survey |
| Excluded participant 7 | Did not complete the social-network survey |

References

1. Parkinson, C., Kleinbaum, A. M. & Wheatley, T. Similar neural responses predict friendship. *Nat. Commun.* **9**, 332 (2018).

2. Hyon, R., Kleinbaum, A. M. & Parkinson, C. Social network proximity predicts similar trajectories of psychological states: Evidence from multi-voxel spatiotemporal dynamics. *NeuroImage* **216**, 116492 (2020).
