# Supplementary material for: In-degree centrality in a social network is linked to coordinated neural activity
Source: Nat Commun. 2022 Mar 2;13:1118. doi: 10.1038/s41467-022-28432-3 (PMC8891270; doi:10.1038/s41467-022-28432-3)
Supplement: Supplementary file 3 — Reporting Summary [file 41467_2022_28432_MOESM3_ESM.pdf]

nature portfolio

Corresponding author(s): Elisa C. Baek
Carolyn Parkinson

Last updated by author(s): November 16, 2021

Reporting Summary

Nature Portfolio wishes to improve the reproducibility of the work that we publish. This form provides structure for consistency and transparency in reporting. For further information on Nature Portfolio policies, see our Editorial Policies and the Editorial Policy Checklist.

Statistics

For all statistical analyses, confirm that the following items are present in the figure legend, table legend, main text, or Methods section.

n/a	Confirmed	
☐	☒	The exact sample size (n) for each experimental group/condition, given as a discrete number and unit of measurement
☐	☒	A statement on whether measurements were taken from distinct samples or whether the same sample was measured repeatedly
☐	☒	The statistical test(s) used AND whether they are one- or two-sided<br>*Only common tests should be described solely by name; describe more complex techniques in the Methods section.*
☐	☒	A description of all covariates tested
☐	☒	A description of any assumptions or corrections, such as tests of normality and adjustment for multiple comparisons
☐	☒	A full description of the statistical parameters including central tendency (e.g. means) or other basic estimates (e.g. regression coefficient) AND variation (e.g. standard deviation) or associated estimates of uncertainty (e.g. confidence intervals)
☐	☒	For null hypothesis testing, the test statistic (e.g. F, t, r) with confidence intervals, effect sizes, degrees of freedom and P value noted<br>*Give P values as exact values whenever suitable.*
☒	☐	For Bayesian analysis, information on the choice of priors and Markov chain Monte Carlo settings
☐	☒	For hierarchical and complex designs, identification of the appropriate level for tests and full reporting of outcomes
☐	☒	Estimates of effect sizes (e.g. Cohen's d, Pearson's r), indicating how they were calculated

Our web collection on statistics for biologists contains articles on many of the points above.

Software and code

Policy information about availability of computer code

Data collection	fMRI data were collected using a 3T Siemens Prisma scanner with a 32-channel coil.
Data analysis	We used fMRIPrep version 1.4.0 for processing of our fMRI data, which included the use of following software: ANTs 2.1.0 and FSL 6.0.<br><br>We calculated inter-subject correlations using SciPy 1.5.3 in Python 3.7.<br><br>Statistical analyses were conducted in R 3.6.1. The social-network data was analyzed using igraph package 1.2.4. GLMs and mixed-effects models were conducted using emmeans 1.4.3.01, lme4 1.1-23, and lmerTest 3.1.0 packages.<br><br>The custom code that we used in the manuscript is available at Zenodo with the accession code 10.5281/zenodo.5711372 [https://zenodo.org/record/5711372].

For manuscripts utilizing custom algorithms or software that are central to the research but not yet described in published literature, software must be made available to editors and reviewers. We strongly encourage code deposition in a community repository (e.g. GitHub). See the Nature Portfolio guidelines for submitting code & software for further information.

Data

Policy information about availability of data

All manuscripts must include a data availability statement. This statement should provide the following information, where applicable:

- Accession codes, unique identifiers, or web links for publicly available datasets
- A description of any restrictions on data availability
- For clinical datasets or third party data, please ensure that the statement adheres to our policy

The data are available at Zenodo with the accession code 10.5281/zenodo.5711351 [https://zenodo.org/record/5711351].

Field-specific reporting

Please select the one below that is the best fit for your research. If you are not sure, read the appropriate sections before making your selection.

☐ Life sciences ☒ Behavioural & social sciences ☐ Ecological, evolutionary & environmental sciences

For a reference copy of the document with all sections, see nature.com/documents/nr-reporting-summary-flat.pdf

Behavioural & social sciences study design

All studies must disclose on these points even when the disclosure is negative.

Study description	This is a quantitative study involving tests for associations between fMRI and social network data.
Research sample	The study research sample consists of first-year undergraduate students living in two different residential communities of first-year students at a large state university (specifically, at University of California, Los Angeles) in the United States. A total of 63 participants (40 female) between the ages of 18 and 21 (with a mean age of M = 18.19 and a standard deviation of SD = 0.59) were included for all analyses. Given that our key research question asked what features distinguish individuals who are centrally located in their social networks, we sought to recruit individuals living in relatively bounded communities where natural variations in individuals' social network centralities could be observed. Hence, our choice of recruiting individuals living in university residential communities is appropriate for our core scientific research question of interest.
Sampling strategy	We sought to recruit as many participants as possible who were living in the two different residential communities at the University of California, Los Angeles. This resulted in a total of 119 participants who completed our social-network survey, and a total of 63 participants (after exclusions) who were included for the fMRI analyses. Our final N size of 63 participants for all analyses is larger than previously published studies investigating the relationships between ISCs and behavioral traits (e.g., Parkinson, Kleinbaum & Wheatley, Nature Comms, 2018; Finn et al., Nature Comms, 2018).
Data collection	The social-network survey was conducted online via participants' computers, and no researchers were present during data collection. Neuroimaging data were collected using a 3T Siemens Prisma scanner with a 32-channel coil, and two researchers were present for all data analysis (due to the safety protocol of the fMRI scanning center). While the researchers were aware of the general hypotheses that were being tested in the study, the researchers were not aware of participants' social-network centralities during data collection. All participants provided informed consent for the social-network survey and the neuroimaging study in accordance with the Institutional Review Board of the University of California, Los Angeles. Participants were compensated in $15 for completing the survey for completing the social-network survey and $50 for completing the neuroimaging study.
Timing	The fMRI data collection occurred between September and early November of 2019, during the subjects' first year at the university. The social-network survey was administered during December 2019 and January 2020.
Data exclusions	A total of 70 participants from the two residential communities participated in the neuroimaging portion of our study. We excluded four subjects from the fMRI data that we analyzed; two subjects had excessive movement in more than half of the scan, one subject fell asleep during half of the scan, and one subject did not complete the scan. Four fMRI subjects did not complete the social-network survey, including one who had already been excluded based on the aforementioned fMRI-based exclusion criteria. Exclusion criteria were pre-established.
Non-participation	One student in the fMRI part of the study ended the scanning session early.
Randomization	Participants were not allocated to experimental groups.

Reporting for specific materials, systems and methods

We require information from authors about some types of materials, experimental systems and methods used in many studies. Here, indicate whether each material, system or method listed is relevant to your study. If you are not sure if a list item applies to your research, read the appropriate section before selecting a response.

Materials & experimental systems

n/a	Involved in the study
☒	☐ Antibodies
☒	☐ Eukaryotic cell lines
☒	☐ Palaeontology and archaeology
☒	☐ Animals and other organisms
☐	☒ Human research participants
☒	☐ Clinical data
☒	☐ Dual use research of concern

Methods

n/a	Involved in the study
☒	☐ ChIP-seq
☒	☐ Flow cytometry
☐	☒ MRI-based neuroimaging

Human research participants

Policy information about studies involving human research participants

Population characteristics: See above.

Recruitment: Participants living in two residential communities of first-year dormitories were recruited via email. One potential concern for self-selection bias could be that highly-central participants may be more likely to participate in the neuroimaging portion of the study. However, a comparison of the distributions of in-degree centralities in the fMRI samples and the full social-network sample (see Fig. S1) suggests that we obtained neuroimaging data from the full possible range of in-degree centralities and that the distributions of in-degree centralities in the full sample and in the subset of the sample who completed the fMRI study, mitigating these concerns.

Ethics oversight: Institutional Review Board of the University of California, Los Angeles.

Note that full information on the approval of the study protocol must also be provided in the manuscript.

Magnetic resonance imaging

Experimental design

Design type: Task fMRI during viewing of naturalistic stimuli

Design specifications: Participants watched 14 different videos in the fMRI scanner. The fMRI study was divided into four runs and lasted approximately 60 minutes in total.

Behavioral performance measures: Given that participants were instructed to passively view the stimuli, no behavioral performance measures were collected.

Acquisition

Imaging type(s): functional, structural

Field strength: 3T

Sequence & imaging parameters: The participants were scanned using a 3T Siemens Prisma scanner with a 32-channel coil. Functional images were recorded using an echo-planar sequence (with echo time = 37 ms, repetition time = 800 ms, voxel size = 2.0 mm × 2.0 mm × 2.0 mm, matrix size = 104 × 104 mm, field of view = 208 mm, slice thickness = 2.0 mm, multi-band acceleration factor = 8, and 72 interleaved slices with no gap). A black screen was included at the beginning (with duration = 8 seconds) and the end (duration = 20 seconds) of each run to allow the BOLD signal to stabilize. We also acquired high-resolution T1-weighted (T1w) images (with echo time = 2.48 ms, repetition time = 1,900 ms, voxel size = 1.0 mm × 1.0 mm × 1.00 mm, matrix size = 256 × 256 mm, field of view = 256 mm, slice thickness = 1.0 mm, and 208 interleaved slices with 0.5 mm gap) for coregistration and normalization. We attached adhesive tape to the head coil in the MRI scanner and applied it across the participants' foreheads, which has been shown to significantly reduce head motion.

Area of acquisition: A whole brain scan was used.

Diffusion MRI: ☐ Used ☒ Not used

Preprocessing

Preprocessing software: We used fMRIPrep version 1.4.0 for the data processing of our fMRI data. We have taken the descriptions of anatomical and functional data preprocessing that begins in the next paragraph from the recommended boilerplate text that is generated by fMRIPrep and released under a CC0 license, with the intention that researchers reuse the text to facilitate clear and consistent descriptions of preprocessing steps, thereby enhancing the reproducibility of studies.
For each subject, the T1-weighted (T1w) image was corrected for intensity non-uniformity (INU) with N4BiasFieldCorrection, distributed with ANTs 2.1.0, and used as T1w-reference throughout the workflow. Brain tissue segmentation of cerebrospinal fluid (CSF), white matter (WM) and gray matter (GM) was performed on the brain-extracted T1w using FSL fast. Volume-

based spatial normalization to the ICBM 152 Nonlinear Asymmetrical template version 2009c (MNI152NLin2009cAsym) was performed through nonlinear registration with antsRegistration (ANTs 2.1.0).
For each of the four BOLD runs per participant, the following preprocessing was performed. First, a reference volume and its skull-stripped version were generated using a custom methodology of fMRIPrep. The BOLD reference was then coregistered to the T1w reference using FSL flirt with the boundary-based registration cost function. The coregistration was configured with nine degrees of freedom to account for distortions remaining in the BOLD reference. Head-motion parameters with respect to the BOLD reference (transformation matrices, and six corresponding rotation and translation parameters) were estimated before any spatiotemporal filtering using FSL mcflirt. Automatic removal of motion artifacts using independent component analysis (ICA–AROMA) was performed on the preprocessed BOLD on MNI space time series after removal of non-steady state volumes and spatial smoothing with an isotropic, Gaussian kernel of 6mm FWHM (full-width half-maximum). The BOLD time series were then resampled to the MNI152Nlin2009cAsym standard space.
The following 10 confounding variables generated by fMRIPrep were included as nuisance regressors: global signals extracted from within the cerebrospinal fluid, white matter, and whole-brain masks, framewise displacement, three translational motion parameters, and three rotational motion parameters.

Normalization	Volume-based spatial normalization to the ICBM 152 Nonlinear Asymmetrical template version 2009c (MNI152NLin2009cAsym) was performed through nonlinear registration with antsRegistration (ANTs 2.1.0).
Normalization template	ICBM 152 Nonlinear Asymmetrical template version 2009c (MNI152NLin2009cAsym)
Noise and artifact removal	Head-motion parameters with respect to the BOLD reference (transformation matrices, and six corresponding rotation and translation parameters) were estimated before any spatiotemporal filtering using FSL mcflirt. Automatic removal of motion artifacts using independent component analysis (ICA–AROMA) was performed on the preprocessed BOLD on MNI space time series after removal of non-steady state volumes and spatial smoothing with an isotropic, Gaussian kernel of 6mm FWHM (full-width half-maximum). The following 10 confounding variables generated by fMRIPrep were included as nuisance regressors: global signals extracted from within the cerebrospinal fluid, white matter, and whole-brain masks, framewise displacement, three translational motion parameters, and three rotational motion parameters.
Volume censoring	Non-steady state volumes were removed for all subjects prior to analysis. We also accounted for motion artifacts using the techniques outlined in "Noise and artifact removal" above.

Statistical modeling & inference

Model type and settings	We calculated inter-subject correlations (ISCs) of time series of neural responses that were measured with fMRI to capture shared neural responses across subjects during the processing of naturalistic stimuli (see Fig. 1). First, we extracted the mean-response time series across the entire video-viewing task from both (1) each of the 200 cortical parcels in the 200-parcel version of the Schaefer et al. (2018) parcellation scheme and (2) 14 subcortical parcels in the Harvard-Oxford subcortical atlas. This resulted in a total of 214 brain regions across the whole brain. For each of the 1,952 unique pairs of participants (i.e., dyads) in our fMRI sample, we then computed the Pearson correlation between the dyad members' time series of neural responses for each cortical parcel. This yields one correlation coefficient per unique dyad for each brain parcel. We then used fit linear mixed-effects models with crossed random effects to test our hypotheses.
Effect(s) tested	We tested the relationships between ISCs and individuals' in-degree centralities.

Specify type of analysis: ☒ Whole brain ☐ ROI-based ☐ Both

Statistic type for inference (See Eklund et al. 2016)	As described in the "Model type and settings" field above, our analyses compare responses within each of 214 anatomically-defined brain regions, and thus are not impacted by the concerns that the Eklund et al. (2016) paper raised regarding inflated false-positive rates in fMRI inferences for spatial extent.<br><br>Correction for multiple comparisons across brain regions was implemented using False-Discovery Rate (FDR) correction (as specified in the "Correction" field below).
Correction	We used FDR correction for all analyses.

Models & analysis

n/a	Involved in the study
☒	☐ Functional and/or effective connectivity
☒	☐ Graph analysis
☒	☐ Multivariate modeling or predictive analysis
